# Effect of transcutaneous acupoint electrical stimulation on propofol sedation: an electroencephalogram analysis of patients undergoing pituitary adenomas resection

**DOI:** 10.1186/s12906-016-1008-1

**Published:** 2016-01-27

**Authors:** Xing Liu, Jing Wang, Baoguo Wang, Ying Hua Wang, Qinglei Teng, Jiaqing Yan, Shuangyan Wang, You Wan

**Affiliations:** 1 Department of Anesthesiology, Beijing Sanbo Brain Hospital, Capital Medical University, Beijing, 100093 China; 2 Department of Neurobiology, Capital Medical University, Beijing, 100069 China; 3 Center for Collaboration and Innovation in Brain and Learning Sciences, Beijing Normal University, Beijing, 100875 China; 4 State Key Laboratory of Cognitive Neuroscience and Learning & IDG/Mc Govern Institute for Brain Research, Beijing Normal University, Beijing, 100875 China; 5 Institute of Electrical Engineering, Yanshan University, Qinhuangdao, 066004 China; 6 Neuroscience Research Institute, Key Lab for Neuroscience, Peking University Health Science Center, Beijing, 100191 China

**Keywords:** Anesthesia, Transcutaneous acupoint electrical stimulation (TAES), Electroencephalogram (EGG), Power spectrum, Pituitary adenoma

## Abstract

**Background:**

Transcutaneous acupoint electrical stimulation (TAES) as a needleless acupuncture has the same effect like traditional manual acupuncture. The combination of TAES and anesthesia has been proved valid in enhancing the anesthetic effects but its mechanisms are still not clear.

**Methods:**

In this study, we investigated the effect of TAES on anesthesia with an electroencephalogram (EEG) oscillation analysis on surgery patients anesthetized with propofol, a widely-used anesthetic in clinical practice. EEG was continuously recorded during light and deep propofol sedation (target-controlled infusion set at 1.0 and 3.0 μg/mL) in ten surgery patients with pituitary tumor excision. Each concentration of propofol was maintained for 6 min and TAES was given at 2–4 min. The changes in EEG power spectrum at different frequency bands (delta, theta, alpha, beta, and gamma) and the coherence of different EEG channels were analyzed.

**Results:**

Our result showed that, after TAES application, the EEG power increased at alpha and beta bands in light sedation of propofol, but reduced at delta and beta bands in deep propofol sedation (*p* < 0.001). In addition, the EEG oscillation analysis showed an enhancement of synchronization at low frequencies and a decline in synchronization at high frequencies between different EEG channels in either light or deep propofol sedation.

**Conclusions:**

Our study showed evidence suggested that TAES may have different effects on propofol under light and deep sedation. TAES could enhance the sedative effect of propofol at low concentration but reduce the sedative effect of propofol at high concentration.

## Background

Transcutaneous acupoint electric stimulation (TAES), or “needleless acupuncture”, is an easy and non-invasive alternative to needle-based electro-acupuncture (EA). It combines the advantages of both acupuncture and transcutaneous electrical nerve stimulation by pasting electrode pads on the acupoints instead of piercing the skin with needles. Several studies indicated that intraoperative TAES could enhance the sedative effect of propofol, a widely-used sedative anesthetic [1–3], In addition, it could reduce the opioids consumption and the incidence of anesthesia-related side-effects, while improve the quality of recovery from anesthesia [4, 5]. Our recent study also suggested that TAES may exert analgesic effect, and the sufentanil consumption was significantly reduced during craniotomy [6]. Electroacupuncture stimulation at ST36 and PC6 has been reported to significantly deepen the sedation level of general anaesthesia [7]. These results suggested that acupuncture and related techniques may have both analgesic and sedative effects. Nevertheless, the combination of TAES and anesthetic has been reported to be benefit, but it is still unclear how acupuncture works in propofol-induced deep or light sedation.

Electroencephalography (EEG) is a sensitive method for measuring the brain activities and is widely applied to monitor the depth of anesthesia. Previous researches showed that activity of EEG changed under anesthesia, power of alpha frequency band (8–12 Hz), especially the frontal alpha, was increased under propofol [8, 9] and beta, gamma frequency band was varies [8]. In addition, disrupted coherence of EEG activity was considered as the leading underlying mechanism of anesthesia [10, 11]. Evidence indicates that TAES or acupuncture could induce the changes of EEG activity. During acupuncture, activity of alpha and theta oscillations of EEG in human being increased [12]. After TAES, the power of theta frequency band was decreased [13]. These results suggested that TAES may modulate the activity and coherence of EEG to improve the sedition under anesthesia [13, 14]. In the present study, we investigated the effect of TAES on propofol anesthesia with an EEG oscillation analysis in patients undergoing pituitary adenoma resection.

## Methods

### Patient selection and clinical procedures

This study was approved by the ethics review board of the Beijing Sanbo Brain Hospital (2013121101) and registered in the Chinese Clinical Trial Registry (registration number: ChiCTR-TRC-13004051). All participants provided their written informed consent and consent to publish the individual and identifiable patient details before being enrolled in this study. Inclusion criteria were as follows: (1) The age of patients should range from 18 to 65 years; (2) Patients without gender limited; (3) The Body Mass Index (BMI) of patients should range from 18 to 30 Kg/m^2^; (4) Patients meet the standard of American Society of Anesthesiology (ASA), Physical Status matain ASA I-II; (5) All patients signed their written informed consent. Exclusion criteria included: (1) Patients had a history of needleless acupuncture within 6 months; (2) Patients in lactation or pregnant; (3) Patients involved in other clinical trial within nearly 4 weeks; (4) Patients took sedatives and analgesics for a long-term, and have been addicted or alcoholics; (6) Patients with extreme anxiety fear, non-cooperation or communication barriers during the test. A total of ten patients scheduled for pituitary tumor excision and met with all above criteria were enrolled in this study during October 2013 to June 2014. All participants received standard pre-anesthesia assessments, and were tested with normal hearing and urine toxicology to exclude other potential factors, which might interact with propofol or confound the EEG adversely.

Participants were fasted for at least 8 h before the procedure. The heart rate of patients was monitored with an electrocardiogram, oxygen saturation through pulse oximetry, respiration and expiratory carbon dioxide with capnography, and blood pressure through non-invasive cuff to ensure the patients’ safety. There were at least three anesthesiologists involved in each study: one was in charge of the medical management of the subject during the study, the second handled the propofol administration, and the third accomplished EEG recording. When the patient became apneic, the first anesthesiologist assisted breathing with bag/mask ventilation. A phenylephrine infusion was applied to maintain mean arterial pressure above the specific level determined from the patients’ baseline measurement.

### Experimental design and procedure

**Fig. 1:**
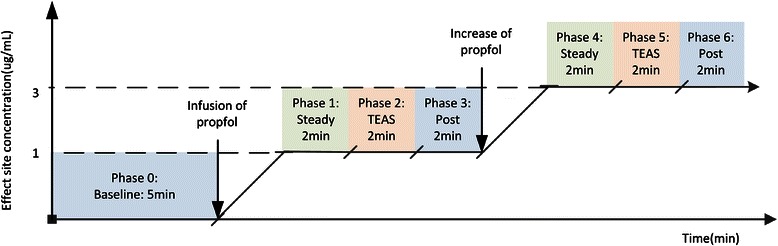
Experimental paradigm. A computer-controlled infusion method was used to achieve propofol target effect-site concentrations of 1 and 3 μg/mL. The target effect-site concentration level was measured for 6 min and then increased to 3 μg/mL. Every 6-min observation period was divided into three phases, and each phase lasted for 2 min. TAES was administrated at the phases two and five, respectively

The experiment paradigm was shown in Fig. 1. Before propofol infusion was started, we recorded the EEG for about 5 min as a baseline when the patient was kept in a conscious, eye-closed and calm situations, this phase was named as phase 0. Then we used a computer-controlled infusion to achieve propofol target effect-site concentrations of 1 μg/mL and then up to 3 μg/mL. Propofol was administered as target-controlled infusion (TCI) based on the pharmacokinetic model by Marsh et al. [15], and the target plasma concentration of sufentanil was set at a certain value (1 or 3 μg/mL) during the whole anesthesia. The concentration level on each target effect-site was maintained for 6 min, then divided each 6 min into three phases (2 min/phase): phase 1–3 (1 μg/mL propofol), and phase 4–6 (3 μg/mL propofol), respectively. TAES were applied in the phase two and phase five.

### TAES

TAES was applied to the acupoints of Hégǔ (LI 4), Wàiguān (TE 5), Zúsānlǐ (ST 36) and Qiūxū (GB 40) on the left side of patient in phase two and five, respectively. The stimulation was applied by the HANS acupoint nerve stimulator (HANS 200A, Nanjing Jisheng Medical Technology Co., Ltd., Nanjing, China) with a dense-disperse frequency of 2/100 Hz (alternated once every 3 s; 0.6 ms at 2 Hz and 0.2 ms at 100 Hz). EEG was recorded in the whole procedure. We defined the whole procedure into three states: state one represents the basal state including phase 0; state two represents low-concentration of propofol (1 μg/mL) including phases one, two, and three (phase 1 is the basal state of phases 2 and 3); state three represents high-concentrationμg/mL of propofol (3 μg/mL) including phases four, five, six (phase 4 is the basal state of phases 5 and 6).

### EEG recordings

Scalp EEG electrodes were positioned at Fp1, Fp2, Fz3, F4, C3, C4, Cz, P3, P4, O1, O2, F7, F8, T3, T4, T5, and T6 (EEG, international 10–20 system); all channels were referenced to A1, A2 (bilateral Mastoid). Electrode impedances were reduced to below 5 kΩ prior to data collection. EEG signals were collected using the Nicolet One EEG-64 device (Nicolet Corp., USA) with a sampling frequency of 1024 Hz. The signals were band-passed at 1.6–45 Hz to avoid baseline drift and high frequency noise.

### Power spectrum analysis

The power spectrum of EEG signals was estimated with a customized procedure as our previous reports [16, 17]. Considering the EEG spectrum should be relatively stable during the short time of each phase (2 min), we used the following method to reduce the abnormal variance in the power spectrum:

1. EEG data were segmented into epochs of 12 s;
2. For each epoch, (1) Perform the Morlet wavelet transform with the wavelet central angle frequency of 6 (ω0 = 6) at frequency band 2.0 Hz to 45 Hz, with a frequency resolution of 0.5 Hz; (2) EEG could have little chance of sudden change during anesthesia. Therefore, we reasonably treat the abrupt change in EEG energy as induced by artifacts. A common fluctuation range of wavelet energy at a particular frequency is within 1 uV. So in this study 1 uV is chosen as the threshold for removing artifacts. Then for each frequency, outliers in corresponding wavelet coefficients which has a standard deviation (SD) larger than 1 μV was removed with a threshold of mean ± SD; (3) For each frequency, repeat step 2.2 with remaining coefficient, until the SD is less than 1 μV, or removed values exceeds a ratio of 20 %;
3. For each frequency, the mean coefficients of all epochs are considered as the power of that frequency.

### EEG coherence analysis

We estimated the magnitude squared coherence between each pair of channels using Welch’s overlapped averaged phaseogram method [18]. The method was described as follows:

For two time series x(n) and y(n), estimate the power spectral density (PSD) by the discrete Fourier transform

$$ {P}_{xx}\left(\omega \right)=\frac{1}{2\pi }{\displaystyle \sum_{m=-\infty}^{\infty }}{R}_{xx}(m){e}^{-j\omega m} $$

and

$$ {P}_{yy}\left(\omega \right)=\frac{1}{2\pi }{\displaystyle \sum_{m=-\infty}^{\infty }}{R}_{yy}(m){e}^{-j\omega m} $$

Then the magnitude-squared coherence between the two signals is given by

$$ {C}_{xy}\left(\omega \right)=\frac{{\left|{P}_{xx}\left(\omega \right){P}_{yy}^{*}\left(\omega \right)\right|}^2}{P_{xx}\left(\omega \right){P}_{yy}\left(\omega \right)} $$

where $*$ denotes the conjugate of a complex number.

$$ \left(\omega \right)=\frac{1}{2\pi }{\displaystyle \sum_{m=-\infty}^{\infty }}{R}_{xx}(m){e}^{-j\omega m} $$

and

$$ {P}_{yy}\left(\omega \right)=\frac{1}{2\pi }{\displaystyle \sum_{m=-\infty}^{\infty }}{R}_{yy}(m){e}^{-j\omega m} $$

Then the magnitude-squared coherence between the two signals is given by

$$ {C}_{xy}\left(\omega \right)=\frac{{\left|{P}_{xx}\left(\omega \right){P}_{yy}^{*}\left(\omega \right)\right|}^2}{P_{xx}\left(\omega \right){P}_{yy}\left(\omega \right)} $$

where * denotes the conjugate of a complex number.

We calculated the coherence index of every channel before and after TAES at each band.

### Statistical analysis

Statistical analysis was performed using SAS 9.0 software (SAS Institute Inc., Cary, NC, USA). The power spectral data before and after TAES were analyzed by paired-sample *t*-test. And the power spectral data from different concentration level of propofol were analyzed by One-Way ANOVA. The further analysis used Dunnett-*t* test, and phase 0 as a control group. Paired-sample *t*-test was also applied to analyze the coherence index before and after TAES at each band for the same channel. A p value of < 0.05 was considered to be statistically significant.

## Results

### Effect of propofol on the EEG power

**Fig. 2:**
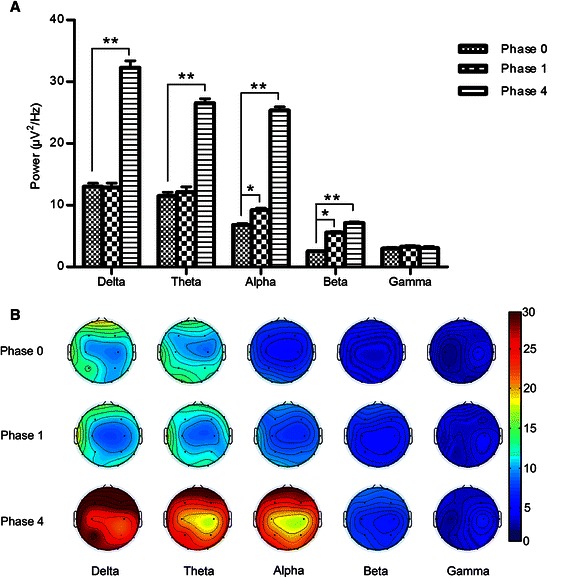
Effect of propofol concentration on the EEG power at different frequency bands. **a**. The EEG power spectrum at each frequency band. **b**. Topoplot of EEG power at each frequency band. Phase 0: before propofol infusion, phase 1: 1 μg/mL propofol, phase 4: 3 μg/mL propofol. Data were color-coded and plotted at the corresponding position on the scalp. ^**^ *p* < 0.001, and ^*^ *p* < 0.05 vs the phase 0

To investigate the effect of propofol on the ongoing brain activities, we calculated the averaged absolute spontaneous EEG powers at each frequency band for each recording session (Fig. 2). One-way ANOVA analysis showed significant difference at delta (F_(2, 158)_, = 187.411, *p* < 0.001), theta (F_(2, 158)_ = 130.379, *p* < 0.001), alpha (F_(2, 158)_ = 742.112, *p* < 0.001), and beta (F_(2, 158)_ = 243.857, *p* < 0.001) bands, but not at gamma band (F_(2, 158)_ = 0.528, *p* = 0.59). Further analysis with Dunnett *t*-test revealed that the power of both alpha and beta frequency oscillations increased significantly at phase one compared with phase 0 (*p* < 0.001), while the power of other bands showed no significant change. In addition, the powers of all of alpha, beta, delta, and theta bands increased significantly at phase four (*p* < 0.001), while the power of gamma band showed no significant changes compared with phase 0.

Furthermore, we analyzed the power changes of different bands in each channel of different phases. Compared with the powers of each band in the corresponding channel of phase 0, the power of alpha band in channels O2 and P4 increased significantly (alpha band: F_(2, 8)_ = 121.778, *p* <0.05), and the power of beta band in all channels increased significantly in phase 1 (F_(2,8)_ = 46.388, *p* < 0.001,). And the power of delta, theta, alpha, and beta bands in all channels increased significantly in phase four (delta band: F_(2, 8)_ = 187.411, *p* < 0.001; theta band: F_(2, 8)_ = 130.379, *p* < 0.001; alpha band: F_(2,8)_ = 742.112, *p* < 0.001; beta band: F_(2, 8)_ = 243.857, *p* < 0.001).

### Effect of TAES on EEG power at different concentrations of propofol

**Fig. 3:**
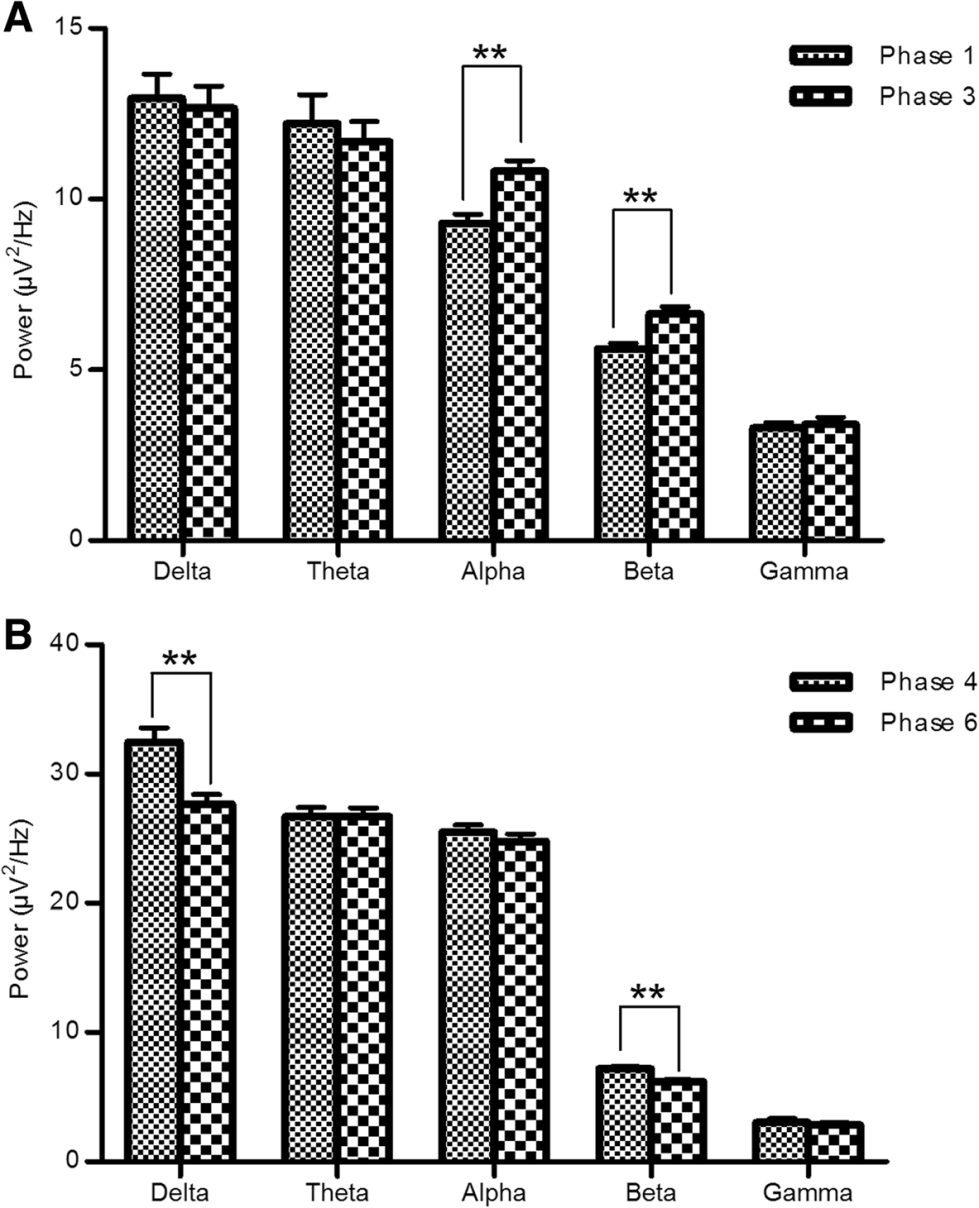
Effect of TAES on the changes of EEG power induced at different propofol concentrations. **a**. EEG power spectrum at propofol concentration of 1 μg/mL; **b**. EEG power spectrum at propofol concentration of 3 μg/mL. In phase two and phase five, TAES were applied. ^**^ *p* < 0.001 vs the phase one or four

**Fig. 4:**
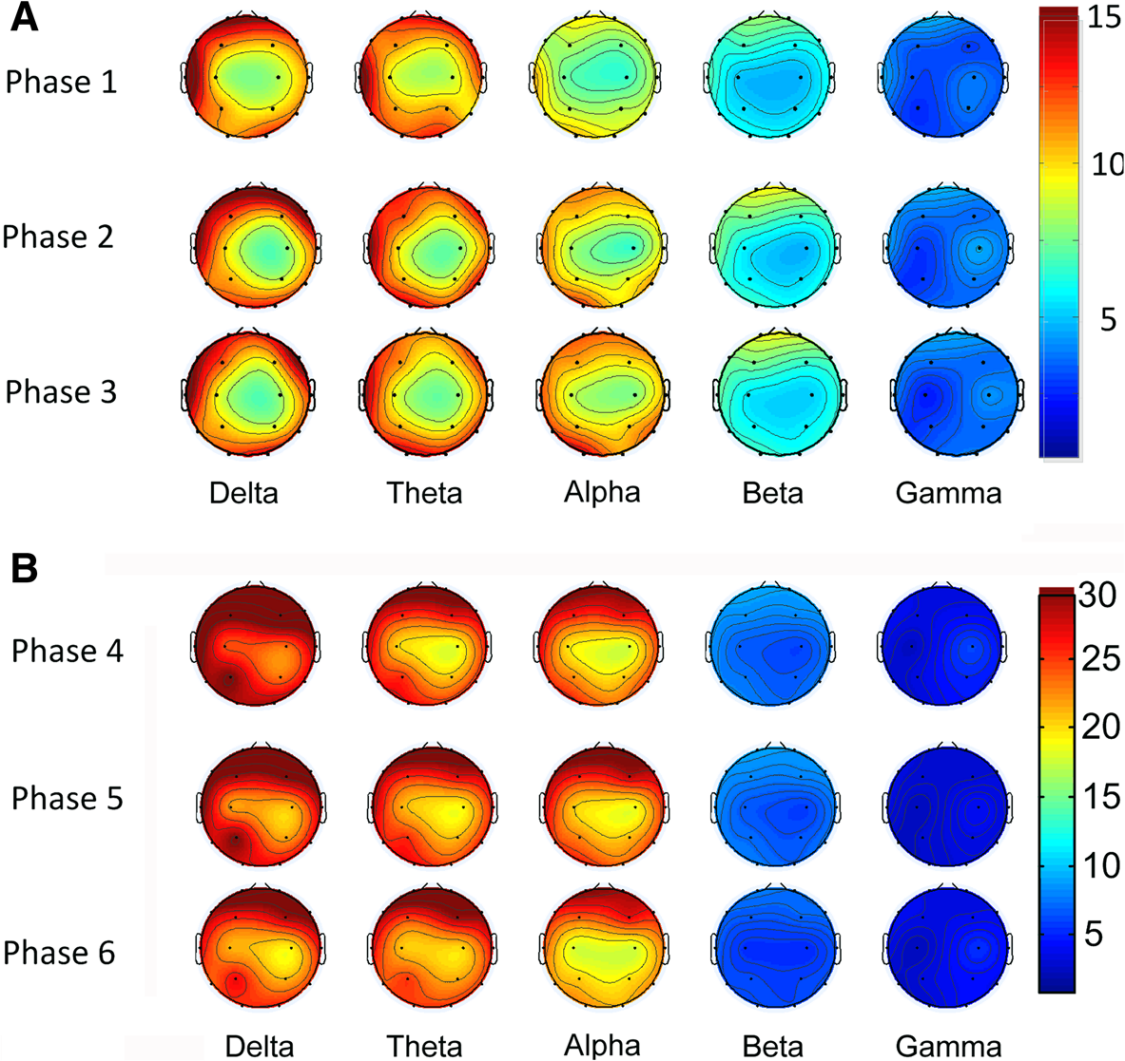
Topoplot of EEG power at different frequency bands during the different phases. **a**: Topoplot of EEG power at the propofol concentration of 1 μg/mL; **b**: Topoplot of EEG power at the propofol concentration of 3 μg/mL TAES was applied in phase two and phase five. Data were color-coded and plotted at the corresponding position on the scalp

We compared and analyzed the averaged absolute power changes of EEG at each frequency band in different phases. Figure 3 showed the effect of TAES on the changes of EEG power in different frequency bands at different concentrations of propofol, we can see an increase at low frequency bands in deep propofol sedation. To observe the main effects of TAES on the propofol-induced EEG power changes, we summarize the EEG power changes in phase one and three and phase four and six, respectively (Fig. 4a and b). Compared with those in phase one, the powers of alpha and beta bands increased significantly (*t* = 7.324, *p* < 0.001; *t* = 9.302, *p* < 0.001) at phase three, whereas the powers of the other bands did not show any significant changes. Compared with those in phase four, the powers of delta and beta bands in phase six showed significant decrease (*t* = 7.819, *p* < 0.001; *t* = 17.312, *p* < 0.001), whereas the powers of the other bands did not show any significant changes.

**Fig. 5:**
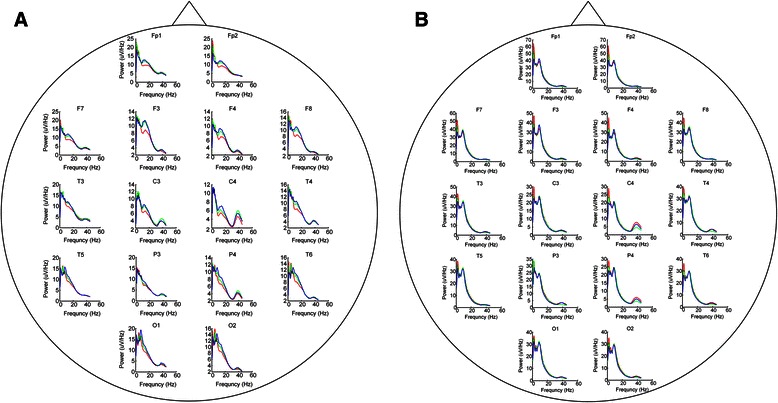
Effect of TAES on the changes of EEG power spectra at each individual channel at different propofol concentrations. **a**: EEG power spectra at propofol concentration of 1 μg/mL; **b**: EEG power spectra at propofol concentration of 3 μg/mL. In each plot, the averaged absolute EEG power before TAES (*red line*), during TAES (*green line*), and after TAES (blue line) is plotted at each electrode

Furthermore, we analyzed the power changes of each band in different phases at each channel (Fig. 5). Compared with that in phase one, the power of alpha band in phase three was among 12 of 16 channels, and the power of beta band was among eight of 16 channels, both of them increased significantly (*p* < 0.001, *t* = 9.457), but the power of other bands did not show any significant changes. Compared with that in phase four, the power of beta band in all channels decreased significantly in phase six, except for channel Fp2. The power of theta band in channels O1 and O2, and the power of delta bands in channel T3, T6 and C4 increased significantly (*p* < 0.001, *t* = 7.331). The power of other bands did not show any significant changes in phase 6. The channels that TAES had significant influences on the EEG power at low- and high-concentrations of propofol were summarized in Table 1.

**Table 1:** Channels with significant change (*p* < 0.05) at different frequency bands, induced by TEAS at two propofol concentrations

Propofol concentration	Frequency band				
	Delta	Theta	Alpha	Beta	Gama
1 μg/mL			O1 O2P4	C3 O1 F7 T5 Fp2 F4 C4 P4 O2 F8 T6	
3 μg/mL	T3 C4T6	O1 O2		Fp1F3P3O1F7T3T5FP2F4C4P4O2F8T4T6	

### Effects of TAES on EEG coherence among different channels at different concentrations of propofol

**Fig. 6:**
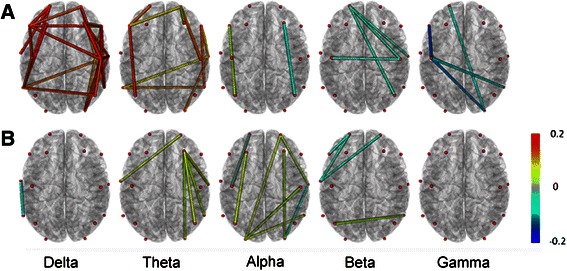
The effect of TAES on synchronization among EEG channels at different frequency bands at different propofol concentrations. **a**: Synchronization among EEG channels at propofol concentration of 1 μg/mL; **b**: Synchronization among EEG channels at propofol concentration of 3 μg/mL. Red nodes indicate electrodes projected onto the cortex. Lines between nodes indicate a significant change in synchronization between the two channels before and after TAES. The color of lines represents the strength of synchronization. The red color indicates an increase in synchronization, and the blue indictaed a decrease

We used magnitude squared coherence to estimate the correlation index between different EEG channels in each band before and after TAES at propofol target effect-site concentrations of 1 μg/mL and 3 μg/mL. Then we used paired-*t* test to analyze the changes of the correlation indices. The discrepancy and the extent of coherence changes of different channels in each band at different propofol concentrations were presented in Fig. 6. The following effects can been seen : Firstly, the synchronization between each pair of channels increased in low-frequency oscillations (delta, theta and alpha), but the synchronism in high-frequency oscillations (beta and gamma) decreased at 1 μg/mL propofol. The synchronization increased in theta and alpha bands at 3 μg/mL propofol, while decreased in beta band, and did not show any significant changes in the rest of the bands; Secondly, the synchronization in ipsilateral hemisphere was stronger than that in bilateral hemispheres at 1 μg/mL propofol. However, it did not show any significant discrepancies between two hemispheres; Thirdly, the synchronization in right hemisphere was stronger than that in left hemisphere at 3 μg/mL propofol.

## Discussion

We adopted a self-control design to compare the changes of brain oscillations before and after TAES in patients undergoing pituitary adenoma resection. The individual difference of EEG data was large. The self-control design can minimize the interference of the individual difference to the results. We used a computer-controlled infusion to achieve stable propofol target effect-site concentrations of 1 μg/mL and 3 μg/mL before and after TAES, respectively. Bonhomme et al. reported that the objects become slightly drowsy at 0.5 μg/mL propofol, inarticulate and sluggish at 1.5 μg/mL, and irresponsive at all at 3.5 μg/mL [19]. Purdon et al. identified two states, one where subjects had a nonzero probability of response to auditory stimulated another where subjects were unconscious with a zero probability of response [9]. Then, some researchers defined upper two states as propofol-induced unconsciousness trough-max (TM) and peak-max (PM), respectively [20]. Furthermore, Akeju’s team found that the neurophysiological signatures were stably maintained over changing propofol effect-site concentrations: approximately 1 to 2 μg/mL for TM and approximately 3 to 5 μg/mL for PM [20]. Thus, we defined 1 μg/mL and 3 μg/mL propofol as light and deep sedation respectively in present study. It not only made it easy to investigate the effect of TAES in a stable neurophysiological state, but also reduced the dose of propofol used for the research.

### Brain oscillation at low- and high-concentrations of propofol

Compared with baseline, the power of alpha and beta oscillations had a significant increase at 1 μg/mL propofol, and power of delta, theta, alpha, and beta significantly increased at 3 μg/mL propofol. Numerous studies have investigated the effect of propofol on EEG, and propofol might exhibit anesthetic effect by potentiating GABA_A_ receptors. The effects on macroscopic dynamics were noticeable in the EEG, which contained several stereotyped patterns during maintenance of propofol anesthesia. These patterns were like that powers in (0.5–4 Hz) delta range increase in light anesthetic level [21, 22]; with the increasing concentration, an alpha (~10 Hz) rhythm [23–25] is coherent across frontal cortex; powers in alpha range then became smaller and theta or delta powers become dominant in deeper levels. With further deeper levels, burst suppression, an alternation between bursts of high-voltage activity and phases of flat EEG was lasting for several seconds [26, 27]. Some researchers investigated the change of EEG power at the loss of consciousness and sedation induced by propofol, a significant increase of beta and alpha bands were observed at sedation of propofol, corresponding to 15–25 Hz and 12–15 Hz, respectively. Additionally, they noticed an enhancement in delta, alpha, and theta power were noticed during propofol induced loss of consciousness [22]. Our results were not completely concordant with pervious study. The reason might be the different propofol concentration used that leading to the different level of sedation, or TAES might influence the brain oscillation, since all patients accepted TAES before the increase in popofol concentration.

### TAES modulation on brain oscillation in light and deep propofol sedation

The validity of TAES in anesthesia was controversial. Some researchers argued that acupuncture was “only” a placebo procedure based on sensory input. However, we observed significant changes of the ongoing power spectra in different frequency oscillations ranging from delta to beta band except for the gamma band.

It is known that alpha oscillation mainly serves as a top-down controlled inhibitory mechanism. Beta oscillation may be involved in the maintenance of the current sensorimotor or cognitive state, and the extraordinary enhancement of beta oscillation may result in an abnormal persistence of the current situation and a deterioration of flexible behaviors and cognitive controls [28]. Theta oscillation serves as an essential network-level role in hippocampal learning and memory. For example, theta oscillations promote plasticity [29] and support memory processes requiring interregional signal integration [30–32]. Conversely, suppression of the theta rhythm impairs learning and memory [33–35]. Delta-band oscillation is more often seen to be related to deep-sleep states and compromise of neuronal function [36]. The latter findings support the belief that low-frequency oscillations might actually influence in active processing [37]. For our result, the brain oscillations induced by TAES in light and deep propofol sedation were different. The power increase in light propofol sedation following TAES intervention occurred at alpha and beta bands, while reduction of power at delta and beta bands was in deep propofol sedation.

As mentioned before, the alpha power has become a reverse measure of activation [38–40]. The beta oscillation might be related to deterioration of cognitive control. The power increases of alpha and beta bands after TAES in propofol sedation might indicate that TAES could strengthen the sedation effect of propofol. The power of delta and beta oscillation was significantly induced after TAES, especially for beta oscillation at 3 μg/mL propofol. The decrease in beta oscillation occurred at all channels. Elevated endogenous GABA levels could cause the elevation of beta power [41, 42]. Electro acupuncture may induce release of endogenous endomorphins that activate μ opioid receptors in GABA-nergic neurons to suppress the release of GABA [43]. Taken together, it is conceivable that the decreased power of beta oscillations in our results might reflect the inhibition of GABAergic interneurons by TAES.

More importantly, we found an enhancement of synchronization at low frequency and a decline in synchronization at high frequency between different channels after TAES, and at different propofol concentrations. Synchronous rhythms represent a vital mechanism for expressing temporal coordination of neural activity in the brain wide network. Coherent oscillations are generated by many generally neuronal synchrony. It may contribute to well-timed coordination and communication between neural populations simultaneously engaged in a cognitive process. It is well known that slow waves oscillations are the electrophysiological correlate of millions of neurons switching between up and down states. The large slow waves may link to decreases in effective connectivity, which presence of widespread cortical disability between up and down states during early NREM sleep [44, 45]. The high frequency oscillations like beta and gamma may play an important role in integrating the unity of conscious perception [46]. It has been accepted that low-frequency oscillations might be involved in the integration of information across widely spatial distribution of neural assemblies and high-frequency oscillations distributed over a more limited topographic area. In our study, we found that the synchronization of low frequency (delta, theta) oscillation occurred widely across brain areas, while the coherence of high frequency (beta, gamma) occurred within more limited areas. Taken together, we speculated that TAES exerted antinociceptive effect by modulation of the power and coherence between different channels, which disturbed the cortex excitability and effective connectivity.

### The deficiency of this article and the future directions of research

There are still some limitations in this view, including limited samples. In the future studies, we will enlarge the sample size to solve this problem. Moreover, there is a washout phase in the self-control design. Although we did not find the conclusive evidence about corporation effect of TAES for 2 min, the post effect of TAES should be taken into consideration when analyze and discuss the EEG changes at 3 μg/mL propofol.

## Conclusion

Changes in EEG signature induced by TAES under light or deep sedation were different. TAES might strengthen the effect of propofol during light sedation, whereas it might have an antagonism to propofol in deep sedation. TAES may exert antinociceptive effect by modulating the power and coherence between different channels, which disturbed the cortex excitability and effective connectivity. However, it is difficult for us to simply come to the conclusion that whether TAES is beneficial or harmful in propofol anesthesia, and a large cohort studies are still needed to further clarify the potential mechanisms of TAES.

## Abbreviations

TEAS: Transcutaneous acupoint electrical stimulation
EGG: Electroencephalogram
EA: Electro-acupuncture
ASA: American society of anesthesiology
PM: Peak-max
TM: Trough-max
